# Oral Health-Related Quality of Life and Traumatic Dental Injuries in Young Permanent Incisors in Brazilian Schoolchildren: A Multilevel Approach

**DOI:** 10.1371/journal.pone.0135369

**Published:** 2015-08-19

**Authors:** Fernanda Bartolomeo Freire-Maia, Sheyla Márcia Auad, Mauro Henrique Nogueira Guimarães de Abreu, Fernanda Sardenberg, Milene Torres Martins, Saul Martins Paiva, Isabela Almeida Pordeus, Míriam Pimenta Vale

**Affiliations:** 1 Department of Paediatric Dentistry and Orthodontics, Universidade Federal de Minas Gerais, Belo Horizonte, Minas Gerais, Brazil; 2 Department of Community and Preventive Dentistry, Universidade Federal de Minas Gerais, Belo Horizonte, Minas Gerais, Brazil; The Ohio State University, UNITED STATES

## Abstract

**Background:**

Traumatic dental injury (TDI) during childhood may negatively impact the quality of life of children.

**Objective:**

To describe the association of oral health-related quality of life (OHRQoL) and domains (oral symptons, functional limitation, emotional- and social-well-being) of children with individual and contextual variables.

**Methods:**

A cross-sectional study was performed using a representative sample of 1,201 schoolchildren, 8–10 years-old, from public and private schools of Belo Horizonte, Brazil. The CPQ_8–10_ was used to assess OHRQoL, dichotomized in low and high impact. Sociodemographic information was collected through questionnaires to parents. Children were examined at schools, using the Andreasen criteria. Individual variables were gender, age, number of residents in home, parents/caregivers’ level of education, family income, and TDI (dichotomized into without trauma/mild trauma and severe trauma). Dental caries and malocclusion were considered co-variables. Contextual variables were the Social Vulnerability Index and type of school. Ethical approval and consent forms were obtained. Data were analyzed using SPSS for Windows 19.0 and HLM 6.06, including frequency distribution, chi-squared test and multilevel approach (p < 0.05).

**Results:**

The prevalence of a negative impact on OHRQoL in children with severe trauma was 55.9%. The TDI negatively impacted emotional and social domains of OHRQoL. A multilevel analysis revealed a significant difference in OHRQoL according to the type of school and showed that 16% of the total variance was due to contextual characteristics (p < 0.001; ICC = 0.16). The negative impact on OHRQoL was higher in girls (p = 0.009), younger children (p = 0.023), with severe TDI (p = 0.014), those from public schools (p = 0.017) and whose parents had a lower education level (p = 0.001).

**Conclusion:**

Severe trauma impacts OHRQoL on emotional and social domains. Contextual dimensions add information to individual variability to explain higher impact, emphasizing socioeconomic inequalities.

## Introduction

Oral health problems may impact children’ daily lives [1–2]. In addition to the clinical measures of disease, it is necessary to document the impact of these oral health problems on their quality of life [3].

The measures of quality of life have a very important role in clinical practice in terms of identifying needs, selecting best therapies, monitoring patients’ progress and helping clinicians to understand the magnitude of benefits that come with the treatment of oral conditions [3]. Many assessment instruments have emerged for measuring the influence of oral conditions on the lives of individuals, more recently called oral health-related quality of life measures (OHRQoL). One of these instruments, the Child Perceptions Questionnaire 8–10 (CPQ_8–10_) was developed in Canada [4] and has been cross-culturally adapted and validated for use in Brazilian children [5].

Moreover, human behavior is not only influenced by well-being and clinical and individual factors but also by socioeconomic conditions and interactions with the environment [6–7]. People in the same environment are subject to common contextual influences [8–9] that may exert an influence on their quality of life and could alter the effect of individual variables.

Traumatic dental injury (TDI) is a common oral disorder in children, which is a distressing experience on a physical level, but may also have an effect on their emotional and psychological levels, presenting a negative impact on their quality of life [1]. The fracture of an anterior tooth can affect the behavior of a child, their progress in school [10], their daily life [1], and that of their family [11]. TDI is presently on the increase in dental clinics and appears with more frequency among children and adolescents than among adults, because of their exposure to sports and games [12]. In Brazil, the prevalence of TDI in permanent teeth, ranges from 9.4% to 58.6% and reports of its influence on quality of life vary greatly because of different methodologies used in studies [13].

Many studies that assess the relationship between OHRQoL and TDI in permanent teeth date from the last five years and included children aged 11–14. Nearly half of the articles were Brazil-based studies [14] and show that TDI results in a severe negative impact on OHRQoL of children. However, despite the fact that children between 8–10 years of age appear to be the most prone to injuries, with the peak occurrence at 9 years of age both in boys and girls [15–16], this age group has not been extensively researched. Therefore, the aim of this study was to verify the association of OHRQoL and domains with individual and contextual variables in this specific age group. The hypothesis to be tested is that children with TDI experience a greater impact on their OHRQoL.

## Materials and Methods

### Study population

This study used a cross-sectional design and was carried out in Belo Horizonte, which is the capital of the state of Minas Gerais (southeastern Brazil), from April to October 2010. The city is geographically divided into nine administrative districts and has 2,375,151 inhabitants [17]. The target population was a representative sample of 1,201 schoolchildren aged 8–10 years old, randomly selected from an initial population of 97,487 children registered at private and public schools, through a multistage sampling method.

The present study was part of a previous cross-sectional survey in which other outcomes were measured, as dental caries and malocclusion [18–19]. The present sample size of 1,201 children provided a 95% confidence interval and a power of 99% in detecting the frequency of impact on quality of life among children with dental trauma (43%) and without dental trauma (24%), based on a previous study [20]. The sample size was based on binomial distribution, since the outcome variable (OHRQoL) was dichotomized.

The inclusion criteria were: children aged 8–10 years, the presence of upper and lower permanent incisors in the oral cavity and previous authorization from parents/caregivers and the child. The exclusion criteria were the use of a fixed orthodontic appliance, since it impedes the best clinical examination [18].

The calibration exercise consisted of two steps, one theoretical and the other practical. The theoretical step involved a discussion of the criteria for the diagnosis of TDI, caries and malocclusion with three gold standards [18, 19, 21]. Caries and malocclusion were used as co-variables. For the practical step, two examiners who were previously calibrated examined 70 children (5% of the sample). Cohen kappa values for interexaminer agreement ranged from 0.71 to 1.00. Fifty children were reexamined after two weeks to assess intraexaminer agreement, for which Cohen kappa values were 0.90 to 1.00. The Cohen kappa agreement was satisfactory to excellent for all clinical conditions [22].

A pilot study was carried out at a convenience school prior to the data collection process. Children in the pilot study were not included in the main sample. The results of the pilot study indicated no need to change the proposed methods.

### Data collection

A questionnaire containing questions on sociodemographic and socioeconomic characteristics was sent to parents/caregivers of each selected children. The type of school (public or private) and the Social Vulnerability Index (SVI) were used as socioeconomic indicators [11, 22, 23]. In Brazil, children from higher socioeconomic conditions generally attend private schools unlike children from lower socioeconomic conditions, who mainly attend public schools [24, 25]. The SVI measures social exclusion in the city of Belo Horizonte. Children usually live near their schools and study in a social environmental similar to that of their homes. Therefore, the SVI was based on the address of each school [22]. This index uses twenty variables based on quantifying access to housing, education, income, jobs, legal assistance, health and nutrition. Its values range from 0 to 1, with higher values indicating worse community conditions and a greater vulnerability to social exclusion in the community in question. The scores for each administrative district of Belo Horizonte were calculated in a previous study carried out by [26]. The SVI classified schools into five different groups/classes. For the statistical analyses, the SVI was grouped into two categories: “high social vulnerability” (Classes I and II) and “low social vulnerability” (Classes III–V) [11, 18].

Impact on OHRQoL was measured using the Brazilian version of the CPQ_8–10_, through interviews with each child in a private room at school, prior to the clinical examination. The items of CPQ_8–10_ address the frequency of events in the previous four weeks. This instrument is made up of 25 items distributed among four domains: OS-oral symptoms (five items), FL-functional limitations (five items), EWB-emotional well-being (five items), and SWB-social well-being (10 items). The response options are: “Never” = 0; “Once or twice” = 1; “Sometimes” = 2; “Often” = 3; and “Very often or almost every day” = 4. The outcome variables were the total score of CPQ_8–10_ and the score for each of its four domains. The total score and domains were dichotomized in the high cluster and low cluster impact on OHRQoL, divided by conglomerates, based on a two-step cluster using a log-likelihood distance measure [1].

Clinical examination was performed by two calibrated pediatric dentists (FS and MTM) at schools, during daytime hours. The examiners were seated in front of the participant, and used a clinical mirror and wooden spatulas to complete the examination. The examiners used appropriate individual cross-infection protection equipment, and all instruments and necessary materials were packaged and sterilized. The dental exam was limited to visual examination.

Traumatic dental injury in the permanent incisors was recorded based on the criteria proposed by [21]. The diagnostic criteria were recorded as follows: no trauma; enamel fracture only; enamel-dentin fracture; complicated fracture (enamel-dentin fracture with pulp involvement); extrusion luxation; lateral luxation; intrusion luxation and avulsion.

**Fig 1:**
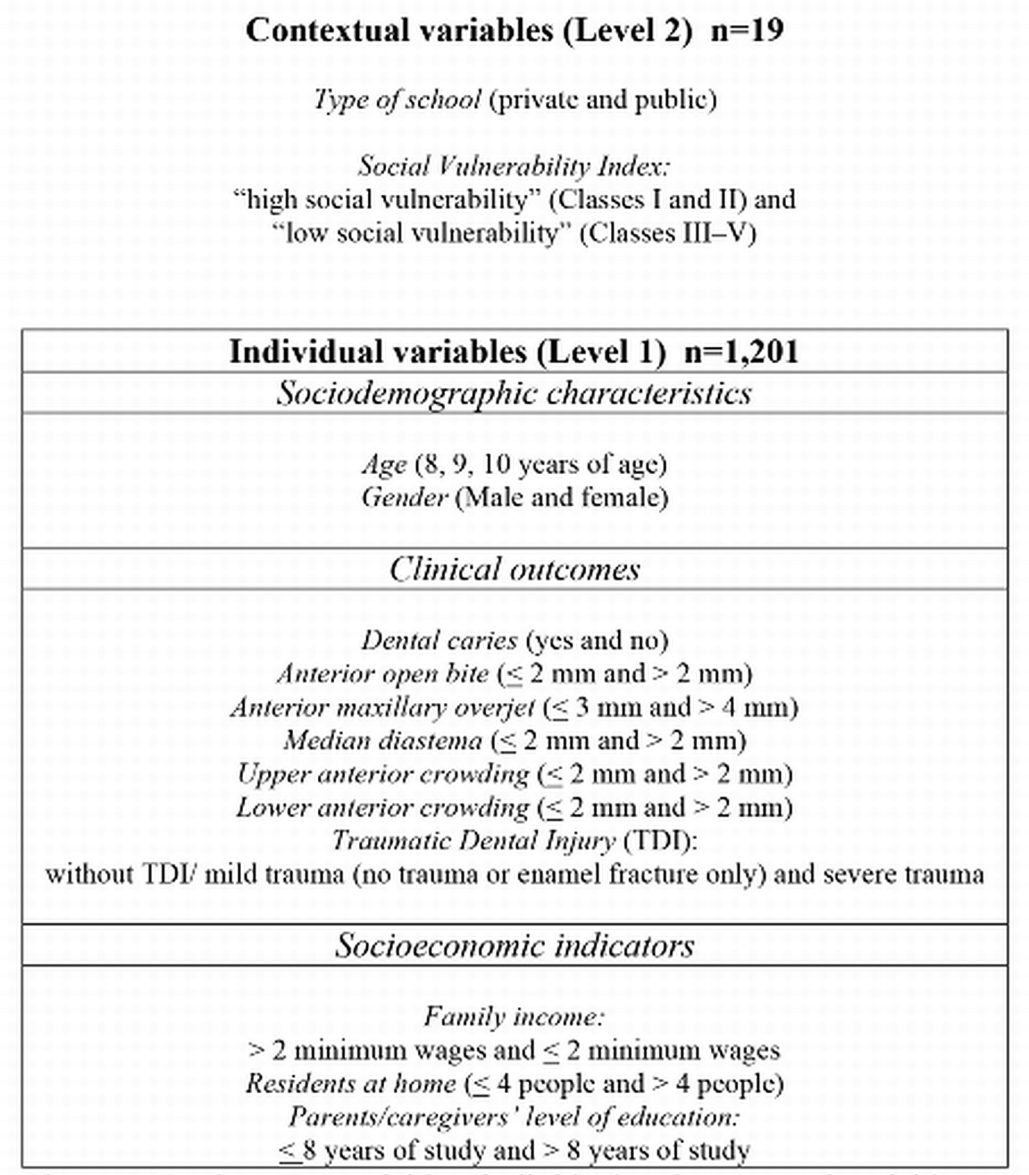
Explanatory variables: individual and contextual variables.

The examination for TDI included only upper and lower permanent incisors, whereas all teeth were examined with regard to the other two oral conditions (dental caries and malocclusion). The World Health Organization criteria were used for diagnosing dental caries. Specific types of malocclusion associated with the anterior teeth were evaluated according to the Dental Aesthetic Index criteria (Fig 1), that is directly related to dental esthetics and dental trauma [18–19].

Two databases (individual and contextual variables) were created with the explanatory variables (Fig 1).

### Ethics statement

This study was approved by the Human Research Ethics Committee of the Federal University of Minas Gerais, Brazil (reg. no. 0465.0.203.000–09). Written statements of consent were read and signed by parents/caregivers and children prior to their participation in the study.

### Statistical analyses

Data analyses involved descriptive statistics (frequency distribution). Normality of data was assessed using the Kolmogorov–Smirnov test (p < 0.001). Bivariate analyses were performed using the Pearson chi-square and Mann-Whitney tests to determine statistically significant associations between dependent (OHRQoL) and independent variables. The level of significance was set at 5%.

Statistical analyses were performed using the Statistical Package for the Social Sciences (SPSS for Windows, version 19.0, SPSS Inc., Chicago, IL, USA) and Hierarchical Linear and Nonlinear Modeling (HLM 6.06 statistical package). SPSS was used for descriptive, bivariate and cluster analyses and to create two databases using individual and contextual variables. These databases were then used in the HLM 6.06 statistical package to perform multilevel analyses [27].

Data was hierarchically structured in two levels: individual variables (Level 1) nested within contextual variables (Level 2) (S1 Dataset). Variables included in Level 1 were gender, age, TDI, dental caries, anterior open bite, anterior maxillary overjet, median diastema, upper anterior crowding and lower anterior crowding, family income, residents in home and parents/caregivers’ level of education. Variables included in Level 2 were type of school and SVI.

Multilevel analyses were used to assess the association of contextual and individual variables with high/low impact on OHRQoL and for each of the domains, and as a control for the potential confounding variables (presence of dental caries and malocclusion).

The multilevel structure of analyses included 1,156 individual variables (Level 1) from 19 schools (Level 2) and was achieved through nonlinear logit link function analyses which used the scheme of fixed effects/random intercept. Parameters were estimated using the restricted maximum likelihood method predictive quasi-likelihood (PQL). A multilevel logistic regression model was constructed. In the first stage a “null model” estimated the basic partition of data variability between the two levels before the inclusion of individual and contextual characteristics were taken into account.

The Level 1 variables were initially incorporated into the model one by one before being tested together (p < 0.20), this was then followed by the test for contextual variables (Level 2), which were incorporated one by one, with the calculation of p-values (Student’s *t*-test). The final model of multilevel analyses was constructed with all the individual variables achieving p < 0.20. In the contextual variables only “type of school” achieved p < 0.20. This strategy allowed for the estimation of the Odds Ratio (OR) among comparison groups and their respective 95% confidence intervals (95% CI). The reliability estimate was used to determine the adequacy of the final multilevel model. All these procedures were performed for each of the four domains, which were considered as a dependent variable each.

## Results

A total of 1,201 children were examined (44.6% boys and 55.4% girls), representing 8–10 year-olds (8 years: 28.1%, 9 years: 35.6% and 10 years: 36.3%). The mean age was 9.8 years (SD = 0.799). The response rate was 83.8% and the main reason for refusal was the child forgetting to ask their parents to sign the statement of informed consent.

The majority of children lived in homes with less than four residents (60.8%) and had parents/caregivers with more than eight years of study (64.3%). Almost half of the families had incomes less or equal to two minimum wages (49.2%). Regarding the respondent’s relationship to the children, 81.1% were mothers, 12.9% were fathers and 6% were others (grandparents, uncles, aunts and tutors), while 76.4% of children studied in a public school (Table 1).

**Table 1:** Frequency distribution of sample (n = 1,201) according to variables: Belo Horizonte, 2010.

Variables	Frequency n (%)
***Individual-level variables***	
**Gender**	
Male	536 (44.6%)
Female	665 (55.4%)
**Age (in years)**	
8	338 (28.1%)
9	427 (35.6%)
10	436 (36.3%)
**TDI**	
Without TDI/mild trauma	1167 (97.2%)
Severe trauma	34 (2.8%)
**Dental caries**	
No	924 (77%)
Yes	276 (23%)
**Anterior open bite**	
≤ 2 mm	1166 (97.1%)
> 2 mm	35 (2.9%)
**Anterior maxillary overjet**	
≤ 3 mm	1105 (92.1%)
> 4 mm	95 (7.9%)
**Median diastema**	
≤ 2 mm	1164 (96.9%)
> 2 mm	37 (3.1%)
**Upper anterior crowding**	
≤ 2 mm	1109 (92.3%)
> 2 mm	92 (7.7%)
**Lower anterior crowding**	
≤ 2 mm	1152 (95.9%)
> 2 mm	49 (4.1%)
**Family income**	
> 2 minimum wages	604 (50.8%)
≤ 2 minimum wages	585 (49.2%)
**Residents in home**	
≤ 4 people	720 (60.8%)
> 4 people	464 (39.2%)
**Parents/caregivers’ level of education**	
> 8 years of study	772 (64.5%)
≤ 8 years of study	425 (35.5%)
***Contextual-level variables***	
**School**	
Private	283 (23.6%)
Public	918 (76.4%)
**Social Vulnerability Index**	
Low (Class III, IV and V)	1058 (88.1%)
High (Class I and II)	143 (11.9%)
***Outcome* (Overall CPQ**_**8–10**_ **)**	
Low	838 (70.5%)
High	351 (29.5%)

Some data is missing for a number of variables. Dental caries: n = 1,200; Residents in home: n = 1,184; Family income: n = 1,189; Parents/caregivers’ level of education: n = 1,197

A total of 1,167 (97.2%) children were “without TDI or had mild trauma” and 34 (2.8%) had “severe trauma”. Enamel trauma only (mild trauma) was found in 135 children (11.2%). Boys had more severe trauma (64.7%) than girls (35.3%), (p = 0.017). A total of 924 children (77.0%) had no untreated dental caries and 7.9% had an anterior maxillary overjet > 4 mm. This was the only variable related to malocclusion statistically associated with TDI (p = 0.005) and impact on OHRQoL (p = 0.010) (Tables 1 and 2).

**Table 2:** Bivariate analyses of individual and contextual variables associated with impact on OHRQoL in children (n = 1,201): Belo Horizonte, 2010.

	OHRQoL cluster			
Variables	Low Impact n(%)	High Impact n(%)	Unadjusted OR (95% CI)	P value
***Individual-level variables***				
**Gender**				
Male	393 (74.0%)	138 (26.0%)	1	
Female	445 (67.6%)	213 (32.4%)	1.36 (1.06–1.76)	0.016^a^
**Age**	9.08 (SD 0.80)			0.029^b^
**TDI**				
Without TDI/mild trauma	823 (71.3%)	332 (28.7%)	1	
Severe trauma	15 (44.1%)	19 (55.9%)	3.14 (1.58–6.25)	0.001^a^
**Dental caries**				
No	685 (75.0%)	228 (25.0%)	1	
Yes	153 (55.6%)	122 (44.4%)	2.40 (1.81–3.17)	<0.001^a^
**Anterior open bite**				
≤ 2 mm	818 (70.9%)	336 (29.1%)	1	
> 2 mm	20 (57.1%)	15 (42.9%)	1.83 (.92–3.61)	0.079 ^a^
**Anterior maxillary overjet**				
≤ 3 mm	782 (71.5%)	311 (28.5%)	1	
> 4 mm	56 (58.9%)	39 (41.1%)	1.75 (1.14–2.69)	0.010 ^a^
**Median diastema**				
≤ 2 mm	811 (70.3%)	342 (29.7%)	1	
> 2 mm	27 (75.0%)	9 (25.0%)	.79 (.37–1.70)	0.546 ^a^
**Upper anterior crowding**				
≤ 2 mm	781 (71.1%)	317 (28.9%)	1	
> 2 mm	57 (62.6%)	34 (37.4%)	1.47 (.94–2.29)	0.088 ^a^
**Lower anterior crowding**				
≤ 2 mm	804 (70.5%)	336 (29.5%)	1	
> 2 mm	34 (69.4%)	15 (30.6%)	1.06 (.57–1.96)	0.864 ^a^
**Family income**				
> 2 minimum wages	475 (79.3%)	124 (20.7%)	1	
≤ 2 minimum wages	353 (61.1%)	225 (38.9%)	2.44 (1.89–3.16)	<0.001^a^
**Residents in home**				
≤ 4	524 (73.5%)	189 (26.5%)	1	
> 4	301 (65.6%)	158 (34.4%)	1.46 (1.13–188)	0.004^a^
**Parents/caregivers’ level of education**				
> 8 years of study	586 (76.6%)	179 (23.4%)	1	
≤ 8 years of study	250 (59.5%)	170 (40.5%)	2.23 (1.72–2.88)	<0.001^a^
***Contextual-level variables***				
**Type of school**				
Private	234 (84.8%)	42 (15.2%)	1	
Public	604 (66.2%)	309 (33.8%)	2.85 (2.00–4.07)	<0.001^a^
**Social Vulnerability index**				
Low (Class III, IV and V)	755 (72.1%)	292 (27.9%)	1	
High (Class I and II)	83 (58.5%)	59 (41.5%)	1.84 (1.28–2.64)	0.001^a^

^a^ Chi-squared test

^b^ Mann-Whitney

OR, Odds Ratio; CI, Confidence Interval

The prevalence of adverse impacts on OHRQoL measured through the o overall CPQ_8–10_ score was 29.5%, and of 41.4%, 27.9%, 23.8% and 19.9% for the OS, FL, EWB and SWB domains, respectively.

In the bivariate analyses of individual and contextual variables associated with impact on OHRQoL, almost all of them were statistically significant, except some types of malocclusion (Table 2). Severe trauma was significantly associated with negative impact on overall quality of life (55.9%) and with three domains: OS (64.7%, OR = 2.67; 95% IC: 1.31–5.46; p = 0.005), SWB (41.2%, OR = 2.93; 95% IC: 1.46–5.90; p = 0.002) and EWB (44.1%, OR = 2.61; 95% IC: 1.31–5.20; p = 0.005) in the bivariate analyses.

The results of the multilevel approach showed the need for the use of a logistic multilevel procedure to analyze the variables. Table 3 shows the final estimation of variance in the multilevel analyses. The “null-model” was statistically significant for the overall CPQ_8–10_ and all domains (p<0.001), and demonstrated significant differences among 19 schools regarding OHRQoL and the four domains. The Intraclass Correlation Coefficient (ICC) showed that 16% of the total variance to overall CPQ_8–10_ was due to contextual characteristics and for domains were: 7% to OS, 19% to FL, 23% to SWB and 16% to EWB.

**Table 3:** Final estimation of variance components in the multilevel analysis (“null-model”).

	Random Effect	Standard Deviation	Variance Component	df	Chi-square	P-value	ICC^a^
**OVERALL OHRQoL**	INTRCPTI, U0	0.79448	0.63120	18	143.22165	<0.001	0.16 = 16%
**OS domain**	INTRCPTI, U0	0.50766	0.25772	18	83.27977	<0.001	0.07 = 7%
**FL domain**	INTRCPTI, U0	0.78070	0.60949	18	119.36268	<0.001	0.19 = 19%
**EWB domain**	INTRCPTI, U0	0.61503	0.37827	18	92.11410	<0.001	0.16 = 16%
**SWB domain**	INTRCPTI, U0	0.96783	0.93670	18	159.94742	<0.001	0.23 = 23%

^a^Intraclass correlation coefficient (ICC): fraction of the total variance that is due to the contextual level

In the initial multilevel models, all individual variables, except residents in home, were statistically significantly associated with an impact on OHRQoL (p < 0.05). For the contextual variables, only the type of school was statistically significantly associated (p < 0.05) (Table 4).

**Table 4:** Bivariate multilevel models for individual and contextual variables associated with OHRQoL in children (n = 1,156): Belo Horizonte, 2010.

Models	Odds Ratio	95% CI	P-value	Reliability estimate
***Individual-level variables***				
**Gender**				0.812
Male	1			
Female	1.36	1.04–1.78	0.025	
**Age**	0.82	0.69–0.99	0.034	0.812
**TDI**				0.812
Without TDI/mild trauma	1			
Severe trauma	2.49	1.21–5.12	0.013	
**Dental caries**				0.813
No	1			
Yes	2.11	1.56–2.87	<0.001	
**Anterior open bite**				0.812
≤ 2 mm	1			
> 2 mm	1.38	0.67–2.84	0.387	
**Anterior maxillary overjet**				0.812
≤ 3 mm	1			
> 4 mm	1.54	0.98–2.43	0.061	
**Median diastema**				0.812
≤ 2 mm	1			
> 2 mm	0.98	0.44–2.19	0.955	
**Upper anterior crowding**				0.812
≤ 2 mm	1			
> 2 mm	1.19	0.74–1.94	0.473	
**Lower anterior crowding**				0.812
≤ 2 mm	1			
> 2 mm	1.35	0.70–2.62	0.377	
**Family income**				0.812
> 2 minimum wages	1			
≤ 2 minimum wages	1.69	1.23–2.32	0.002	
**Residents in home**				0.812
≤ 4	1			
> 4	1.26	0.96–1.66	0.101	
**Parents/caregivers’ level of education**				0.812
> 8 years of study	1			
≤ 8 years of study	1.72	1.27–2.32	0.001	
***Contextual-level variables***				
**Type of school**				0.746
Private	1			
Public	2.67	1.25–5.70	0.015	
**Social Vulnerability Index**				0.812
High (Class I and II)	1			
Low (Class III, IV and V)	1.88	0.47–7.47	0.351	

In the final model of multilevel analyses with total score of impact on OHRQoL, the remaining individual variables were gender, age, parents/caregivers’ level of education, TDI, dental caries and anterior maxillary overjet. The type of school was the only remaining contextual variable (p < 0.05). These individuals and contextual variables were identified as determinants of the negative impact on children’s OHRQoL. Girls had a 1.46-fold greater chance of presenting a high negative impact on OHRQoL and younger children had more chance of a high negative impact (8 years: 34.5%; 9 years: 28.2%; 10 years: 26.0%) (Table 5). Children with severe dental trauma (55.9%) reported more negative impact on OHRQoL than children with dental caries (44.4%) and/or accentuated anterior maxillary overjet (41.1%). Higher proportions were also observed for three domains, when the above conditions were assessed: OS (64.7%, 54.7%, 50.5%, respectively), EWB (44.1%, 38.4%, 33.7%, respectively) and SWB (41.2%, 27.9%, 30.5%, respectively). For the FL domain, children with anterior maxillary overjet > 4mm reported more negative impact on OHRQoL (43.2%) than children with severe trauma (38.2%) and dental caries (37.1%).

**Table 5:** Final multilevel model for individual and contextual variables associated with impact on OHRQoL in children (n = 1,156): Belo Horizonte, 2010.

Models	Odds Ratio	95% CI	P-value	Reliability estimate
**Gender**				0.754
Male	1			
Female	1.46	1.10–1.92	0.009	
**Age (in years)**	0.81	0.67–0.97	0.023	
**Dental caries**				
No	1			
Yes	2.05	1.50–2.80	<0.001	
**Anterior maxillary overjet**				
≤ 3	1			
> 4	1.64	1.03–2.62	0.037	
**TDI**				
Without TDI/mild trauma	1			
Severe trauma	2.54	1.21–5.31	0.014	
**Parents/caregivers’ level of education**				
> 8 years of study	1			
≤ 8 years of study	1.72	1.26–2.35	0.001	
**Type of school**				
Private	1			
Public	2.70	1.22–5.94	0.017	

Representing socioeconomic indicators, parents/caregivers’ level of education (individual levels) remained in the final model, where lower level of education (8 or under years) had a 1.72-fold greater chance of presenting a high negative impact on OHRQoL than higher level of education. In the contextual variables, public school represented a 2.70-fold greater chance of presenting a high negative impact on OHRQoL than private school (Table 5).

As for the domains, a high negative impact on EWB and SWB was associated with gender (p = 0.003; p = 0.045, respectively), severe dental trauma (p = 0.047; p = 0.044, respectively), parents/ caregivers’ level of education (p = 0.002; p = 0.002, respectively) and the type of school (p = 0.007; p = 0.034, respectively). The domains OS and FL were not associated with TDI (Table 6).

**Table 6:** Final multilevel model for individual and contextual variables associated with impact on domains OS, FL, EWB, SWB of OHRQoL in children (n = 1,156): Belo Horizonte, 2010.

Models	Odds Ratio	95% CI	P-value	Reliability estimate
**OS domain**				0.734
**Dental caries**				
No	1			
Yes	2.14	1.59–2.88	<0.001	
**Residents in home**				
≤ 4	1			
> 4	1.45	1.12–1.87	0.005	
**FL Domain**				0.813
**Dental caries**				
No	1			
Yes	1.56	1.14–2.14	0.006	
**Anterior maxillary overjet**				
≤ 3	1			
> 4	1.92	1.22–3.03	0.005	
**Family income**				
> 2 minimum wages	1			
≤ 2 minimum wages	1.59	1.15–2.20	0.006	
**EWB Domain**				0.600
**Gender**				
Male	1			
Female	1.60	1.19–2.15	0.003	
**Dental caries**				
No	1			
Yes	2.45	1.78–3.37	<0.001	
**Anterior maxillary overjet**				
≤ 3	1			
> 4	1.73	1.07–2.81	0.027	
**Lower anterior crowding**				
≤ 2 mm	1			
> 2 mm	2.75	1.44–5.25	0.003	
**TDI**				
Without TDI/mild trauma	1			
Severe trauma	2.12	1.01–4.43	0.047	
**Parents/caregivers’ level of education**				
> 8 years of study	1			
≤ 8 years of study	1.70	1.22–2.34	0.002	
**Type of school**				
Private	1			
Public	2.63	1.37–5.06	0.007	
**SWB Domains**				0.750
**Gender**				
Male	1			
Female	1.38	1.01–1.89	0.045	
**Dental caries**				
No	1			
Yes	1.50	1.06–1.11	0.022	
**Anterior maxillary overjet**				
≤ 3	1			
> 4	1.66	1.00–2.73	0.049	
**TDI**				
Without TDI/mild trauma	1			
Severe trauma	2.18	1.02–4.64	0.044	
**Parents/caregivers’ level of education**				
> 8 years of study	1			
≤ 8 years of study	1.75	1.24–2.46	0.002	
**Type of school**				
Private	1			
Public	2.92	1.10–7.79	0.034	

## Discussion

A reduced prevalence of severe trauma negatively impacted the quality of life of Brazilian 8–10-year-old schoolchildren for the total score of CPQ_8–10_ and for the emotional and social well-being domains, thus confirming the hypothesis of the present study. The significant association of individual and contextual variables and negative impact on OHRQoL were observed in this specific age group, agreeing with [28], in a study on Brazilian preschool children (1–5 years of age). Our results complement and corroborate the findings of studies which found an impact on OHRQoL in preschoolers [29–31] and adolescents with TDI [1, 20, 23, 32–35] and also make an important contribution to the existing literature, because this age group (8–10 years) has not been examined much in the past.

In a longitudinal study assessing severe dental trauma among 8–14 years old, children and adolescents reported an impact on the emotional- or social well-being domains [34], similarly to our results. Also, families of adolescents with more severe TDI were more likely to report a negative impact on OHRQoL, affecting family activities and emotions [11]. Also, children with untreated TDI were more likely to experience a negative impact on social well-being, avoided smiling or laughing and were concerned about what other people may think or say [23]. They also reported a negative impact in emotional well-being [35–36].

Functional limitations were not associated, and the OS domain was associated with TDI only in bivariate analyses. An explanation for these results is a possible elapsed time between the occurrence of trauma and the application of the questionnaire.

A previous study carried out in Brazil involving children in the same age group (8–10-year-olds) showed no differences in quality of life among children with TDI or severe TDI when compared with children without TDI; this is contrary to our findings, which may be due to a different methodology (pattern of cutoff points of CPQ_8–10_ and criteria of TDI). However, self-reported dental trauma was shown to be associated with a more negative impact on OHRQoL (p < 0.001) [37]. Others studies have also shown no association between quality of life and TDI in other age groups [38–43] or have found an association only with some specific domains of quality of life [23, 36, 44].

Methodological differences may lead to different results in the impact on quality of life. There is no pattern in the cutoff points used in studies to classify the impact on OHRQoL as present or absent, low or high. Some studies have only used one positive response in the questionnaire to qualify a high impact [20, 23, 32, 36], or the 80th percentile [33], average [45], mean [10, 34–37, 39, 44, 46], median [40] or cluster, all utilizing the score of the CPQ to separate groups [1]. In our study the outcome variable “low or high impact on OHRQoL” was assessed through two-step cluster analyses of CPQ_8–10_, which is a useful tool for identifying profiles associated with multifactorial processes and creating true clusters of OHRQoL, similar to [1]. Another divergent point is the inclusion of mild trauma (enamel trauma) in analyses of TDI. The majority of studies that did not find a relationship with TDI and OHRQoL included enamel trauma (which is not considered a severe trauma) together with more serious traumas which “diluted” the final results [37, 39–40, 44]. As enamel fracture is more prevalent and has very little effect on quality of life, it does not seem to be a problem for children and is not perceived as problematic for individuals or families [11, 41]. On the other hand, severe trauma can affect aesthetics or function, cause pain and interfere with the social lives and well-being of children and their parents [1, 3, 20, 23, 32–35].

Brazil is a country of social inequalities and the present study considered family income, number of residents in home, parents/caregivers’ level of education (Level 1 –individual variables), type of school and SVI (Level 2 –contextual variables) as proxies for socioeconomic status. All these variables were associated with impact on children’s OHRQoL in bivariate analyses (Table 2). However, in the final models of multilevel analysis, only parents/caregivers’ level of education and the type of school remained significant. These variables indicate socioeconomic conditions. On the individual level, lower number of years of education of parents/caregivers, and on the contextual level, attending public schools, were more likely to impact the OHRQoL of children. These are aspects that should be considered in planning public policies. Health and the related quality of life is not just a matter of health policy, but also of social and economic policies [47]. These findings corroborate studies that have already demonstrated a positive relationship between socioeconomic factors and OHRQoL, where being disadvantaged socioeconomically is related to an important decline in quality of life [28, 33, 37, 40, 45, 47]. One possible reason is the limited access to health centers and consequently difficulty receiving treatment for oral problems, worse quality of care received or limitation of people’s opportunities for choice and decision making [28], which may influence their perception of OHRQoL.

The type of school was the only contextual variable included in our study that had a statistically significant association (p < 0.05) with higher impact on OHRQoL and also on the emotional- and social well-being domains in 8–10-year-old schoolchildren. Children attending public schools showed higher severity scores of OHRQoL. As children studying in the same school share common contextual influences, they tend to experience a similar impact on OHRQoL [48]. Children spend a considerable amount of their time in school, and the school environment is therefore of importance for child outcomes and also has an impact on child health and well-being [48]. Furthermore, the role of the school’s social and physical environment as determinants of dental trauma injuries has been well established [49–50]. Schools that are small, safe, with better teacher supervision of children, parental participation and community activities are the healthiest environments and are associated with lower injury rates. Thus, this may influence the impact on OHRQoL in schoolchildren. Furthermore, since schools are pivotal to children’s intellectual, social, and emotional development, school connectedness may be a factor that contributes to their perception of OHRQoL [51]. Clinical conditions such as dental pain [52], TDI [7], dental caries [19], malocclusion [18] and their relation with OHRQoL seemed to vary between schools. Interventions focusing on school context, rather than only on individual factors, may be effective in preventing impact on OHRQoL [48], especially in reducing socioeconomic disadvantages.

The results of this study show variations in the perception on emotional and social domains, which are dependent on aspects relating to individuals and their contexts. Importantly, this conclusion has several implications for paediatric dentistry practice and schools, which was associated contextual variables found. In another study [53], children registered at public schools, from families with low income and with mothers with less years of school education reported significant impact with all domains (OS, FL, EWB and SWB), reinforcing that not only clinical conditions are associated with OHRQoL, but social and environmental variables may interfere in children’s quality of life, agreeing with the present results and previous work [45].

In this study, there was a difference between genders in relation to the impact on the total score of OHRQoL (p < 0.05), and on the emotional and social domains. Although boys were more severely affected by TDI, girls showed more negative effects in their quality of life, a finding that agrees with some authors [33, 40–41, 45]. This association between gender and OHRQoL may be explained by a higher concern of girls with functional and aesthetic-related health issues [33, 40–41, 45]. Evidence was found for marked sex differences in the neural mechanisms underlying emotional processes, and in most cases, suggested that males and females use different strategies during emotional processing [54]. Males and females differ in the recruitment of cerebral networks when processing emotions and different factors including hormonal, chromosomal, environmental are of influence [55].

Another finding of this study is that younger children showed greater negative impact on OHRQoL. Older children may have broader experience with pain and seem better able to manage it due to a larger assortment of coping strategies than younger children [34]. Therefore, prevention through health promotion and the correction of predisposing risk factors should happen during the early mixed dentition period to reduce the prevalence of dental injury and its impact on OHRQoL [15]. Other authors did not find any association between age and OHRQoL [1].

In spite of the significant results of this study, they should be interpreted within the context of some limitations. The study had a cross-sectional design, which made it difficult to evaluate the indicators of risk for OHRQoL. Therefore, longitudinal follow-up is required. However, we observe that cross-sectional studies are important tools for identifying risk factors to be included in further longitudinal assessments. Furthermore, the clinical examinations were performed in the schools, which did not allow the use of X-rays and may have led to underestimation of TDI due to the inability to detect root fractures. Careful attention should be given while analyzing the type of injury because it can vary according to the place where the study is conducted [15]. Schools are not a referential center of dental trauma and we encountered a low prevalence of severe dental trauma. However, this diagnostic procedure allowed us to obtain a larger population-based sample size with an epidemiological nature representative of the city of Belo Horizonte.

The implications from the findings of this study are important. Knowing that severe TDI adversely affects the quality of life of children 8–10 years of age and their emotional and social aspects, it is important to prepare dental professionals to receive and assist the child and their family in such a stressful situation. The procedures to be taken and the possible prognosis must be carefully explained. The proposed treatment should aim to reestablish function and aesthetics, and consequently, oral health, with a later follow-up. Professional of schools (teachers, caregivers) should also be prepared to assist these children and promoting well-being in schools, as 16% of the impact on children’s quality of life is related to the school context, being 23% in the social aspects and 16% in the emotional aspects. Children’ classmates should receive the child in a positive way, avoiding constraints.

In dental conditions that have a strong aesthetic or functional component or cause adverse health consequences, such as severe trauma, the use of normative assessments combined with measures of quality of life should be considered. Moreover, it is important to evaluate contextual variables that might interfere with individual variables in order to promote a reduction in inequalities regarding health.

## Conclusions

Both contextual (type of school) and individual (gender, age, parents/caregivers’ level of education and oral conditions, especially TDI) level factors were significantly associated with the OHRQoL, with emphasis on socioeconomic inequalities. All these factors are relevant to the planning of public health programs and may contribute to the definition of groups with higher levels of need.

## Supporting Information

S1 Dataset Individual and contextual variables (Level 1 and Level 2). (ZIP)
